# Disparities in Diffuse Cortical White Matter Integrity Between Socioeconomic Groups

**DOI:** 10.3389/fnhum.2019.00198

**Published:** 2019-06-12

**Authors:** Danielle Shaked, Daniel K. Leibel, Leslie I. Katzel, Christos Davatzikos, Rao P. Gullapalli, Stephen L. Seliger, Guray Erus, Michele K. Evans, Alan B. Zonderman, Shari R. Waldstein

**Affiliations:** ^1^Department of Psychology, University of Maryland, Baltimore, MD, United States; ^2^Laboratory of Epidemiology and Population Sciences, National Institute on Aging Intramural Research Program, Baltimore, MD, United States; ^3^Division of Gerontology and Geriatric Medicine, University of Maryland School of Medicine, Baltimore, MD, United States; ^4^Geriatric Research Education and Clinical Center, Baltimore VA Medical Center, Baltimore, MD, United States; ^5^Biomedical Image Analysis, Department of Radiology, University of Pennsylvania, Philadelphia, PA, United States; ^6^Department of Diagnostic Radiology, University of Maryland School of Medicine, Baltimore, MD, United States; ^7^Division of Nephrology, Department of Medicine, University of Maryland School of Medicine, Baltimore, MD, United States

**Keywords:** white matter integrity, health disparities, diffusion tensor imaging, socioeconomic status, race, age, neuroanatomical health

## Abstract

There is a growing literature demonstrating a link between lower socioeconomic status (SES) and poorer neuroanatomical health, such as smaller total and regional gray and white matter volumes, as well as greater white matter lesion volumes. Little is known, however, about the relation between SES and white matter integrity. Here we examined the relation between SES and white matter integrity of the brain’s primary cortical regions, and evaluated potential moderating influences of age and self-identified race. Participants were 192 neurologically intact, community-dwelling African American and White adults (mean age = 52 years; 44% male, 60% White, low SES = 52%) from the Healthy Aging in Neighborhoods of Diversity across the Life Span (HANDLS) SCAN study. Participants underwent 3.0-T cranial magnetic resonance imaging. Diffusion tensor imaging was used to estimate regional fractional anisotropy (FA) to quantify the brain’s white matter integrity and trace to capture diffusivity. Multiple regression analyses examined independent and interactive associations of SES, age, and race with FA of the frontal, temporal, parietal, and occipital lobes bilaterally. Sensitivity analyses assessed the influence of several biopsychosocial risk factors on these associations. Exploratory analyses examined these relations with trace and using additional SES indicators. Results indicated there were no significant interactions of SES, age, and race for any region. Individuals with low SES had lower FA in all regions, and higher trace in the right and left frontal, right and left temporal, and left occipital lobes. Findings remained largely unchanged after inclusion of sensitivity variables. Older age was associated with lower FA and greater trace for all regions, except for the right temporal lobe with FA. No main effects were found for race in FA, and Whites had higher trace values in the parietal lobes. Novel findings of this study indicate that relative to the high SES group, low SES was associated with poorer white matter integrity and greater diffusivity. These results may, in part, reflect exposures to various biopsychosocial risk factors experienced by those of lower SES across the lifespan, and may help explain the preponderance of cognitive and functional disparities between socioeconomic groups.

## Introduction

There is a burgeoning literature demonstrating a link between socioeconomic status (SES) and neuroanatomical health. For instance, on average, those lower on the socioeconomic ladder have smaller total (Waldstein et al., 2017) and regional gray and white matter volumes (for reviews see: Hackman and Farah, 2009; McEwen and Gianaros, 2010; Brito and Noble, 2014). Studies have also shown that lower childhood (Murray et al., 2014) and adult SES (Waldstein et al., 2017) are related to greater white matter lesion burden in adults. Little is known however about the relation between SES and white matter integrity, and the present literature is equivocal.

At least two studies found no relation between SES and white matter microstructure (i.e., integrity) in children (Chiang et al., 2011; Jednorog et al., 2012), although one of those studies found that children from higher SES environments were more likely to inherit greater fractional anisotropy (FA) in several brain regions (Chiang et al., 2011). Conversely, at least two other studies found that higher levels of SES in children are associated with greater white matter integrity in several fiber tracts (Ursache and Noble, 2016; Dufford and Kim, 2017). One additional study in children found significant relations between SES and FA in certain white matter tracts, but these results were in the unexpected direction in that higher SES was linked to lower FA (Noble et al., 2013). Although the literature is limited, associations between lower SES in adulthood and poorer white matter integrity have also been found in both neurological disease (Teipel et al., 2009) and non-clinical (Piras et al., 2011; Gianaros et al., 2013; Johnson et al., 2013) adult populations. Inconsistencies in the literature could be due to several factors, such as differences in chronological age, sociodemographic makeup of the samples, and overall white matter maturation across study samples.

The majority of SES-white matter integrity findings have been demonstrated in major, localized white matter fiber tracts, such as the superior longitudinal fasciculus (Gianaros et al., 2013; Noble et al., 2013; Dufford and Kim, 2017) and the cingulum bundle (Noble et al., 2013; Ursache and Noble, 2016; Dufford and Kim, 2017). However, the unique constellation of brain regions affected by SES differs across studies, and there may be regional specificity to the relation between SES and white matter integrity. For instance, white matter integrity of the temporal lobe may be differentially important in the context of SES (Teipel et al., 2009; Piras et al., 2011). More research is needed to determine if relations between SES and FA are uniform to the entire brain, or regionally specific.

Previous studies have also reported associations between self-identified race and brain health endpoints. In the United States, it is well documented that African Americans experience a disproportionate burden of poor clinical brain health compared to other racial/ethnic groups (Harwood and Ownby, 2000; Mozaffarian et al., 2016). Disparities in stroke risk are most pronounced, particularly during middle adulthood, such that African Americans are 3–4 times more likely than Whites to experience stroke by age 45 (Morgenstern et al., 1997). Racial disparities are also found in the frequency and severity of white matter lesions (Liao et al., 1997), as well as prevalence and incidence of Alzheimer’s disease and other forms of dementia (Tang et al., 2001; Demirovic et al., 2003). African Americans also have greater burdens of vascular risk factors than their White counterparts, including obesity (Wang and Beydoun, 2007), diabetes mellitus (LaVeist et al., 2009), and hypertension (Hertz et al., 2005), which may deleteriously affect brain health. Despite evidence for racial disparities across a broad range of brain health outcomes, to our knowledge, relations of self-identified race with white matter integrity have not been examined in community-dwelling samples.

Considerable evidence suggests that aging is associated with deterioration of white matter as demonstrated by decreases in FA (Salat et al., 2005). Age-related decreases in FA have been found to differ by brain region (for a review see Raz and Rodrigue, 2006), such that reductions in white matter FA are generally greater in the frontal white matter compared to the temporal, parietal, and occipital lobes (Head et al., 2004; Salat et al., 2005). Indeed, converging evidence suggests an anterior-posterior gradient of age-related FA decreases (Sullivan and Pfefferbaum, 2006).

Only one study has examined concurrent age-, race- and SES-related differences in white matter microstructure. Johnson et al. (2013) examined associations between SES (as indicated by a composite measure of occupational and educational attainment) and white matter integrity in cognitively normal younger (mean age = 33.3 years) and older adults (mean age = 66.2 years). After adjustment for age, sex, and IQ, they found age-related differences in white matter integrity across a wide range of brain regions. However, among the older adults only, higher SES was associated with greater white matter integrity in three frontal tracts: the right anterior corona radiata and bilateral white matter regions underlying the superior frontal gyri.

No studies have examined interactive relations among SES, race, and age with white matter integrity. This is notable because previous research has shown that these sociodemographic characteristics may have synergistic influences on brain and other health endpoints. For example, racial health disparities cannot be fully explained by SES (Williams et al., 2010), as demonstrated by Waldstein et al. (2017) who found that African Americans of higher SES did not differ from lower SES African Americans with respect to their total brain and white matter lesion volumes. It is also plausible that SES-related brain health disparities are greater at later periods in the adult lifespan, given the relatively high prevalence of age-related diseases among individuals of lower SES (Williams et al., 2010). This is consistent with theories of cumulative disadvantage that have demonstrated an aggregation of inequity throughout the lifespan (O’Rand, 1996; Epel et al., 2018), suggesting that SES-brain disparities may be more profound at older ages.

Several physiological, behavioral, and psychosocial risk factors may be important to consider when investigating the relation between SES and white matter integrity. Risk factors for cardiovascular disease, such as obesity, hypertension, diabetes, cigarette smoking, and systemic inflammation, have a detrimental impact on brain health (Waldstein and Elias, 2015). In an important study conducted by Gianaros et al. (2013), adiposity and smoking status independently mediated the relation between SES and white matter integrity, with high-sensitivity C-reactive protein (CRP) accounting for much of the variance in those meditational paths. This is consistent with the literature showing links between SES and poorer cardiovascular health (Pollitt et al., 2005), and between cardiovascular health and white matter integrity (Wersching et al., 2010; Stanek et al., 2011; Gow et al., 2012). Depressive symptomatology has also been linked to indicators of poor brain health. Previous studies have demonstrated that depression is a risk factor for stroke morbidity and mortality (for a meta-analysis see Pan et al., 2011). Higher rates of depression among individuals with poorer socioeconomic conditions are also well-documented (Hackman et al., 2010), and late life depression is associated with the frequency and intensity of white matter abnormalities (for a review see Herrmann et al., 2008) and changes in white matter microstructure as measured by FA (Yang et al., 2007).

**FIGURE 1:**
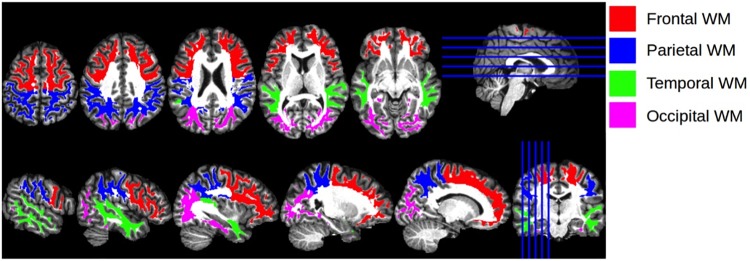
Cortical Regions of Interest. Illustration of the anatomical regions of interest that were used for calculating regional mean fractional anisotropy and trace values. The four regions of interest, encoded with different colors for visualization, are shown overlaid on the T1 atlas image.

Given the prognostic importance of white matter integrity on functional and neurocognitive outcomes (Madden et al., 2009), we examined associations of SES with diffuse white matter integrity, an approach that has not been examined previously. We also examined potential moderating roles of self-identified race and age on the association between SES and FA of the brain’s primary cortical regions, including the right (R) and left (L) frontal, temporal, parietal, and occipital lobes (Figure 1). The methodological approach of using lobar measures of white matter integrity has been used in other contexts (e.g., Voss et al., 2013; Roalf et al., 2015), as it allows researchers to examine general trends across the brain while making fewer comparisons than using all of the brain’s white matter tracts. This methodology can expand the present literature using tract-based approaches.

We also ran sensitivity analyses to examine whether adjustment for several key biomedical (i.e., body mass index [BMI], hypertension, diabetes, CRP), behavioral (i.e., cigarette smoking), and psychosocial (i.e., depressive symptoms) risk factors changed the findings observed in the main analyses, suggesting mediating effects of these co-morbid factors. We chose to examine these risk factors due to their well-documented associations with SES (Kaplan and Keil, 1993; Lorant et al., 2003) and white matter integrity (Burgmans et al., 2010; Gons et al., 2011; Korgaonkar et al., 2011; Stanek et al., 2011; Zhang et al., 2014; Walker et al., 2017). Given that BMI may have non-linear associations with health outcomes (e.g., Laxy et al., 2017), both linear and quadratic BMI were examined. To assess whether overall white matter vascular burden eliminated significant effects, we also ran sensitivity analyses adjusting for whole-brain white matter lesion volume. Finally, exploratory analyses examined (1) models using three different SES indicators on regional FA and (2) independent and interactive relations of SES, race, and age on trace diffusion, which captures diffusion of water across three perpendicular orientations.

## Materials and Methods

### Sample and Participants

Participants were drawn from the Healthy Aging in Neighborhoods of Diversity across the Life Span (HANDLS) SCAN study, an investigation of brain health disparities attributable to race and SES (Waldstein et al., 2017). HANDLS SCAN is an ancillary study of the larger HANDLS investigation, a prospective, epidemiologic study of race- and SES-related health disparities. The design and implementation of the HANDLS parent study has been described previously (Evans et al., 2010). Briefly, the HANDLS sample is a fixed cohort of community-dwelling adults living in 13 neighborhoods (contiguous census tracts) in Baltimore City. The census segments were pre-determined for their likelihood of yielding representative samples of participants who were African American and White, men and women, aged 30–64 years, and with annual household income above and below 125% of the 2004 federal poverty level. HANDLS SCAN data collection overlapped with the first and second follow-up of the parent study. The imaging subsample used in this study is representative of the larger HANDLS study with regards to years of education, poverty status, and sex (*p*’s > 0.05), but is more likely to include White and younger participants relative to the overall study sample (*p*’s < 0.05).

Participants were excluded from the HANDLS parent study if they were (1) outside of the age range of 30–64 years, (2) currently pregnant, (3) within 6 months of active cancer treatment (i.e., chemotherapy, radiation, or biological treatments), (4) diagnosed with AIDS, (5) unable to provide informed consent, (6) unable to provide data for at least five measures, (7) unable to provide valid government-issued identification or were currently without a verifiable address (Evans et al., 2010). In addition to these criteria, HANDLS SCAN excluded participants with a self-reported history of dementia, stroke, transient ischemic attack, other neurological disease (e.g., multiple sclerosis, Parkinson’s disease, or epilepsy), carotid endarterectomy, terminal illness (e.g., metastatic cancer), HIV positive status, or MRI contraindications (e.g., indwelling ferromagnetic material).

The present study’s sample consisted of 192 HANDLS SCAN participants who had complete data for all relevant sociodemographic (SES, race, age, and sex) and diffusion tensor imaging (DTI) data with no incidental clinical findings on MRI.

### Procedure

#### HANDLS

HANDLS investigators recruited participants in each household by performing doorstep interviews, and inviting one or two eligible individuals per household to participate in the study. Successfully recruited and consented individuals were asked to complete an in-home 24-h dietary recall interview and a household survey inquiring about demographic, psychosocial, and physiological information. Participants were then scheduled for additional testing on the mobile research vehicles (MRVs). The Institutional Review Board (IRB) of the National Institute of Environmental Health Services, National Institutes of Health approved the HANDLS study.

#### HANDLS SCAN

HANDLS participants were invited to participate in HANDLS SCAN during their MRV visit. After successfully completing an eligibility screening inventory, participants provided written informed and HIPAA consent in accordance with Declaration of Helsinki. Participants were examined by a physician at the University of Maryland General Clinical Research Center for a brief medical evaluation to identify any acute medical problems since their last HANDLS visit, re-administer the MRI eligibility checklist, review current medications, and assess whether there were any contraindications precluding HANDLS SCAN testing. The subjects underwent MRI acquisition in the Department of Diagnostic Radiology and Nuclear Medicine at the University of Maryland School of Medicine. The IRBs of the University of Maryland, Baltimore and University of Maryland, Baltimore County approved the HANDLS SCAN study. Participants received $50 for their participation.

### Measures

#### Sociodemographic Characteristics

Age (in years), sex (0 = female; 1 = male), poverty status, and education were assessed at study entry (data collection 2004–2009).

HANDLS investigators based their initial recruitment on a division of household income based on 125% of the 2004 federal poverty level. They effectively recruited a similar number of participants above and below the poverty level, as to appropriately represent individuals from low and moderate levels of income. Many HANDLS participants could not estimate their annual incomes, had no way to estimate their overall wealth, and/or were not consistently employed. Because HANDLS does not have an accurate estimate of income, a “poverty status” variable defined as household income above or below 125% of the 2004 federal poverty level adjusted for household size is used. Given the use of poverty status (a dichotomized variable in its original form), it was combined with a dichotomized measure of education to remain parsimonious. While there is controversy in the literature on how to best capture SES, we believe that our sample is best characterized by a composite measure of both education and income, which is consistent with prior recommendations (Adler et al., 1994).

Socioeconomic status was therefore comprised of: (1) dichotomous poverty status (0 = non-poverty; 1 = poverty); and (2) dichotomous years of education (0 = greater than or equal to 12 years; 1 = fewer than 12 years; Waldstein et al., 2017; Shaked et al., 2018, 2019). SES was dichotomized as high and low based on these two measures. Low SES was defined as having low education (<12 years), being below the poverty line, or both. Participants were classified as high SES if they were both living above the poverty level and had ≥12 years of education.

#### Sensitivity Variables

Depressive symptoms, cigarette smoking, BMI, hypertension, diabetes, and inflammation were assessed at the corresponding HANDLS visit. Depressive symptoms were assessed using the Center for Epidemiologic Studies-Depression 20-item scale (Radloff, 1977). Cigarette smoking was assessed via self-report during the medical history assessment (coded as 0 = never used regularly, 1 = ever used regularly). BMI was computed as weight divided by height squared (kg/m^2^) using height and weight obtained via calibrated equipment by a training technician. Hypertension and diabetes were dichotomous variables (coded as 0 = absent, 1 = present). Hypertension was determined by self-reported history, use of anti-hypertensive medications, or resting systolic or diastolic blood pressures ≥ 140 mm Hg or ≥ 90 mm Hg. Diabetes was determined by a fasting blood glucose level of ≥ 126 mg/dl (assessed by standard laboratory methods at Quest Diagnostics in Chantilly, VA, United States^1^), self-reported history, or use of relevant medications. Inflammation was assessed with high-sensitivity CRP (mg/l) levels, which were measured from blood samples by immunoassay at the National Institutes of Aging or Quest Diagnostics using similar equipment and reagents. A HANDLS physician or nurse practitioner documented all clinical diagnostic data after a comprehensive physical examination and medical history.

### Diffusion Tensor Imaging Acquisition and Processing

Cranial magnetic resonance images were acquired using a Siemens Tim-Trio 3.0 Tesla scanner within the Core for Translational Research in Imaging @ Maryland (C-TRIM), part of the Department of Diagnostic Radiology at University of Maryland Baltimore’s School of Medicine. With regards to structural imaging, in addition to the standard brain imaging protocol, which includes axial T1, T2, FLAIR images, a high-resolution axial T1-weighted MPRAGE (TE = 2.32 ms, TR = 1900 ms, TI = 900 ms, flip angle = 9°, resolution = 256 × 256 × 96, FOV = 230 mm, sl. thick. = 0.9 mm) covering the entire brain was acquired. It was used both as an anatomic reference and to extract parameters of regional and whole brain volumes, and cortical thickness.

DTI was obtained using multi-band spin echo EPI sequence with a multi-band acceleration factor of three. Isotropic resolution images were acquired with an in-plane resolution of 2 × 2 mm and 2 mm slice thickness over a 22.4 cm FOV. A total of 66 slices at a TE = 122 ms, TR = 3300 ms, and flip angle = 90° were used. Bipolar diffusion scheme was used to reduce the effect of eddy currents. Diffusion weighting scheme was a 2-shell (*b* = 1000 and 2500 s/mm^2^), optimized for uniform sampling of each shell and non-overlapping diffusion directions of 60 and 120 for each shell, respectively, and 6 b0 volumes. The image acquisition time was 10 min.

The raw diffusion weighted images (DWI) data was denoised using the Joint Linear Minimum Mean Squared Error denoising software (jLMMSE; Tristan-Vega and Aja-Fernandez, 2010). The diffusion tensor images were then reconstructed from denoised DWI data by fitting the tensor using multivariate linear fitting, while also performing motion correction using FSL’ s “eddy correct” tool (Andersson and Sotiropoulos, 2016). FA and trace images were computed from the tensor image for each subject. FA, a widely known method for quantifying white matter integrity that is sensitive to the degree of myelination, density, and organization of white matter, was used to determine the degree of water diffusion directionality within brain tissue. The FA value, which measures the degree of anisotropy of the diffusion at a voxel, is computed from the variance of the average of the three eigenvalues of the diffusion tensor. FA values range from 0 to 1, with 0 reflecting completely unrestricted diffusion, and 1 reflecting completely restricted diffusion. Generally, healthier white matter integrity refers to more restricted diffusion, and thus for the purposes of this study, higher FA values are indicative of healthier white matter integrity. The trace value, which measures diffusivity, is computed by adding the eigenvalues of the diffusion tensor (Jones, 2008). After FA and trace images were computed from the tensor image for each participant, they were aligned to a common template space via deformable registration using a standard DTI template known as EVE (Wakana et al., 2004). In the present study, FA and trace from related cortical white matter subregions were averaged and summed, respectively, to create mean FA and total trace values for the larger brain regions, namely the R and L frontal, parietal, temporal, and occipital lobes. The cortical white matter subregions comprising the larger brain regions were drawn from previous literature (see Roalf et al., 2015).

#### Magnetic-Resonance Imaging-Assessed Lesion Volume

Structural MRI scans were preprocessed by removal of extra-cranial material on T1-weighted image using a multi-atlas registration-based method (Doshi et al., 2013), followed by bias correction (Tustison et al., 2010). A supervised learning based multi-modal lesion segmentation technique was applied to segment ischemic lesions (Zacharaki et al., 2008). The method involved co-registration of T1, T2, and FLAIR scans, histogram normalization to a template image, feature extraction, voxel wise label assignment using a model that was trained on an external training set with manually labeled ground-truth lesion masks, and false-positive elimination. The total white matter lesion volume was calculated for each subject from the segmented lesion mask.

### Analytic Plan

All statistical analyses were performed by the Statistical Package for the Social Sciences (IBM Corp. Released 2017. IBM SPSS Statistics for Windows, Version 25.0. Armonk, NY: IBM: Corp.). Descriptive analyses were conducted to assess means, standard deviations, distributions, and linearity of variables. Main analyses were multiple linear regression to examine independent and interactive associations of age, race, and SES with white matter integrity of the R and L frontal, parietal, temporal, and occipital regions, adjusting for sex. Non-significant (at *p* > 0.05) interaction terms were removed by a backward elimination procedure (see Morrell et al., 1997).

#### Sensitivity and Exploratory Analyses

Subsequent, separate sensitivity analyses were conducted to assess respective contributions of BMI, hypertension, diabetes, CRP, cigarette smoking, depressive symptoms, and whole-brain white matter lesion volume as covariates in the aforementioned models. Due to missing data for depressive symptoms (*n* = 7 missing), CRP (*n* = 12 missing), and cigarette smoking (*n* = 25), data were imputed using a predictive mean matching method with the ‘MICE’ package in R (R Core Team, 2018), resulting in complete samples for all variables.

Using the same multiple regression models, supplemental exploratory analyses examined (1) independent and interactive relations of age, race, and individual SES (i.e., non-composite) indicators including poverty status, continuous education, and dichotomous education (0 = greater than or equal to 12 years/GED; 1 = less than 12 years) with FA; and (2) independent and interactive relations of age, race, and the SES composite with regional trace.

## Results

### Demographics and Variable Characteristics

Table 1 shows demographic data and variable characteristics for the overall sample, and by SES and race. There were significant differences in age, sex, depressive symptoms, and cigarette smoking between those of low and high SES, wherein those of high SES, on average, were older, more likely to be male, have depressive symptoms, and smoke compared to those of low SES. Across race, Whites were, on average, older than African Americans. Those of high SES, on average, had higher FA in the frontal and occipital lobes bilaterally. There were no FA differences across racial groups.

**Table 1:** Demographic and health variables for the overall sample and by SES and race.

	Overall (*N* = 192)	High SES (*n* = 99)	Low SES (*n* = 93)		AA (*n* = 77)	White (*n* = 115)	
Variable	M (SD) or *n* (%)	M (SD) or *n* (%)	M (SD) or *n* (%)	*p*_1_	M (SD) or *n* (%)	M (SD) or *n* (%)	*p*_2_
Age, y	52.03 (9.24)	53.99 (9.60)	49.95 (8.41)	0.002	50.28 (9.87)	53.21 (8.65)	0.031
AA	77 (40.1%)	33 (33.3%)	44 (47.3%)	0.049	–	–	–
Female	107 (55.75)	47 (47.5%)	60 (64.5%)	0.017	46 (59.7%)	61 (53.0%)	0.362
Low SES	95 (48.4%)	–	–	–	44 (57.1%)	49 (42.6%)	0.049
Hypertension	88 (45.8%)	51 (51.5%)	37 (39.8%)	0.104	39 (50.6%)	49 (42.6%)	0.276
Diabetes	30 (15.6%)	18 (18.2%)	12 (12.9%)	0.317	13 (16.9%)	17 (14.8%)	0.696
BMI, kg/m^2^	29.57 (6.43)	29.95 (6.26)	29.16 (6.61)	0.398	29.66 (6.40)	29.50 (6.47)	0.868
CRP, mg/l	5.71 (10.12)	4.81 (7.87)	6.75 (12.36)	0.195	4.98 (7.45)	6.80 (13.12)	0.222
Cigarettes (ever)	139 (72.4%)	67 (67.7%)	72 (77.4%)	0.022	83 (72.2%)	77 (72.7%)	0.549
CES-D score	15.93 (11.49)	14.22 (10.60)	17.74 (12.17)	0.034	16.36 (12.07)	15.29 (10.62)	0.528
WMLV (cc)	1,294 (2,243)	1,210 (1,973)	1,385 (2,513)	0.594	1.188 (1,721)	1,431 (2,850)	0.431
R Frontal, FA	0.235 (0.013)	0.237 (0.011)	0.232 (0.014)	0.006	0.233 (0.013)	0.235 (0.013)	0.306
L Frontal, FA	0.234 (0.014)	0.237 (0.012)	0.232 (0.014)	0.009	0.233 (0.013)	0.235 (0.014)	0.349
R Temporal, FA	0.249 (0.014)	0.250 (0.014)	0.247 (0.014)	0.075	0.248 (0.012)	0.245 (0.015)	0.811
L Temporal, FA	0.241 (0.014)	0.243 (0.013)	0.239 (0.015)	0.064	0.241 (0.012)	0.241 (0.015)	0.849
R Parietal, FA	0.228 (0.015)	0.229 (0.014)	0.227 (0.016)	0.418	0.229 (0.015)	0.227 (0.015)	0.344
L Parietal, FA	0.232 (0.013)	0.233 (0.013)	0.230 (0.014)	0.055	0.231 (0.013)	0.232 (0.014)	0.894
R Occipital, FA	0.205 (0.013)	0.207 (0.013)	0.202 (0.013)	0.010	0.204 (0.011)	0.205 (0.014)	0.694
L Occipital, FA	0.203 (0.014)	0.205 (0.012)	0.200 (0.015)	0.019	0.203 (0.013)	0.204 (0.014)	0.642


*y = years; AA = African American; SES = socioeconomic status; BMI = body mass index; CRP = C-reactive protein; CES-D = Center for Epidemiologic Studies-Depression scale; WMLV = white matter lesion volume; R = right; L = left; FA = fractional anisotropy; *p*_*1*_ = value for the difference between those high and low SES; *p*_*2*_ = value for the difference between African Americans and Whites; independent samples *t*-tests were used for continuous variables (all equal variances assumed) and one-way ANOVAs were used for categorical variables. Fractional anisotropy values range from 0 to 1, with 0 reflecting completely unrestricted diffusion, and 1 reflecting completely restricted diffusion.*

### Regression Analyses

There were no significant interactions between race, SES, or age on any of the cortical white matter regions (all *p*’s > 0.05; see Table 2). There were significant main effects for SES for all regions, wherein individuals with low SES had lower FA in all cortical white matter regions (all *p*’s < 0.05; see Table 3), demonstrating that individuals with lower SES had poorer white matter integrity throughout the entire brain. Significant main effects for age were found for nearly all regions (all *p*’s > 0.05) such that older age was associated with lower FA in the cortical white matter regions, with the exception of the R temporal lobe, β = -0.09, *p* = 0.208, showing that poorer white matter integrity is related to older age almost uniformly across the brain. A main effect for sex was found for the R parietal lobe, wherein men had lower FA in this region, β = -0.16, *p* = 0.029. No significant main effects were found for race (all *p*’s > 0.05).

**Table 2:** Sociodemographic variables and regional fractional anisotropy: full models.

	Outcome (Fractional Anisotropy; *N* = 192)															
	R Frontal		L Frontal		R Temporal		L Temporal		R Parietal		L Parietal		R Occipital		L Occipital	
Predictor	β	*sr^2^*	β	*sr^2^*	β	*sr^2^*	β	*sr^2^*	β	*sr^2^*	β	*sr^2^*	β	*sr^2^*	β	*sr^2^*
SES∗Age∗Race	–0.02	0.000	–0.15	0.000	0.20	0.001	0.01	0.000	0.58	0.005	0.32	0.001	–0.18	0.000	0.22	0.001
Age∗Race	–0.54	0.006	–0.27	0.001	–0.42	0.003	–0.38	0.003	–0.44	0.004	–0.48	0.004	–0.27	0.002	–0.54	0.006
Age∗SES	–0.18	0.001	0.08	0.000	–0.38	0.003	–0.10	0.000	–0.41	0.003	–0.24	0.001	–0.15	0.000	–0.44	0.004
Race∗SES	–0.08	0.000	0.20	0.000	–0.29	0.001	–0.09	0.000	–0.58	0.004	–0.32	0.001	0.11	0.000	–0.38	0.002
Age	–0.21	0.013	–0.27*	0.020	–0.01	0.000	–0.14	0.005	–0.20	0.011	–0.21	0.012	–0.21	0.013	–0.11	0.003
Race	0.49	0.004	0.15	0.000	–0.44	0.003	0.42	0.003	0.45	0.003	0.43	0.003	0.26	0.001	0.57	0.005
SES	–0.07	0.000	–0.38	0.002	0.24	0.001	–0.08	0.000	0.24	0.001	0.01	0.000	–0.09	0.000	0.26	0.001
Sex	–0.13	0.016	–0.08	0.005	–0.14	0.017	–0.14	0.017	–0.16*	0.023	–0.09	0.008	–0.09	0.007	–0.12	0.014


*SES = socioeconomic status; R = right; L = left. Full models reflect the models that include all interactions, prior to the backward elimination procedure. ^∗^*p* < 0.05. Data reflect standardized regression coefficients (β) and semipartial correlations squared (*sr^2^*).*

**Table 3:** Sociodemographic variables and regional fractional anisotropy: final models.

	Outcome (Fractional Anisotropy; *N* = 192)															
	R Frontal		L Frontal		R Temporal		L Temporal		R Parietal		L Parietal		R Occipital		L Occipital	
Predictor	β	*sr^2^*	β	*sr^2^*	β	*sr^2^*	β	*sr^2^*	β	*sr^2^*	β	*sr^2^*	β	*sr^2^*	β	*sr^2^*
Age	–0.31***	0.088	–0.29***	0.078	–0.09	0.008	–0.18*	0.029	–0.26***	0.062	–0.28***	0.072	–0.28***	0.075	–0.24**	0.052
SES	–0.28***	0.069	–0.25**	0.058	–0.17*	0.026	–0.20**	0.035	–0.15*	0.020	–0.21**	0.041	–0.26***	0.061	–0.24**	0.050
Race	–0.09	0.008	–0.08	0.006	–0.02	0.000	0.01	0.000	0.04	0.002	–0.03	0.001	–0.04	0.002	–0.05	0.002
Sex	–0.13	0.016	–0.07	0.005	–0.14	0.018	–0.13	0.017	–0.16*	0.023	–0.09	0.008	–0.08	.007	–0.12	0.014


*SES = socioeconomic status; R = right; L = left. Final models reflect the models following the backward elimination procedure. ^∗^*p* < 0.05, ^∗∗^*p* < 0.01, ^∗∗∗^*p* < 0.001. Data reflect standardized regression coefficients (β) and semipartial correlations squared (*sr^*2*^*).*

### Sensitivity Analyses

There were no significant main effects of hypertension(range of β = -0.09 to β = -0.02; all *p*’s > 0.05), diabetes (range of β = -0.04 to β = 0.07; all *p*’s > 0.05), linear BMI (range of β = -0.06 to β = -0.01; all *p*’s > 0.05), quadratic BMI (range of β = -0.08 to β = 0.06; all *p*’s > 0.05), CRP (range of β = -0.05 to β = 0.04; all *p*’s > 0.05), cigarette smoking (range of β = -0.06 to β = 0.001; all *p*’s > 0.05), or depressive symptoms (range of β = -0.13 to β = 0.04; all *p*’s > 0.05) on any of the cortical white matter regions. All significant main effects of SES (see Table 4 for associations of SES and regional FA outcomes after adjustment for sensitivity variables), age, and sex described previously remained significant after adjusting for hypertension, diabetes, linear and quadratic BMI, and CRP (all *p*’s < 0.05). The significant association between SES and the R parietal lobe became non-significant following adjustment for depressive symptoms (attenuation to *p* = 0.058) and cigarette smoking (attenuation to *p* = 0.06), although the magnitude of changes in β were small (change from β = -0.15 to β = -0.14 following adjustment for either variable). Adding depressive symptoms and quadratic BMI also rendered main effects of sex with the R frontal and temporal lobes significant (all *p*’s < 0.05), such that relative to women, men had lower FA in these regions.

**Table 4:** Associations of the main effect of SES with regional fractional anisotropy, adjusted for sensitivity variables.

	Outcome (*N* = 192)															
	R Frontal		L Frontal		R Temporal		L Temporal		R Parietal		L Parietal		R Occipital		L Occipital	
Covariate	β	*sr^2^*	β	*sr^2^*	β	*sr^2^*	β	*sr^2^*	β	*sr^2^*	β	*sr^2^*	β	*sr^2^*	β	*sr^2^*
Base model^a^	–0.28***	0.07	–0.25**	0.06	–0.17*	.03	–0.20**	0.04	–0.15*	0.02	–0.21**	0.04	–0.26***	0.06	–0.24**	0.05
Hypertension	–0.28***	0.08	–0.26**	0.06	–0.18*	0.03	–0.20**	0.04	–0.15*	0.02	–0.21**	0.04	–0.26***	0.07	–0.24**	0.05
Diabetes	–0.28***	0.08	–0.25**	0.06	–0.17*	0.03	–0.20**	0.04	–0.15*	0.02	–0.21**	0.04	–0.26***	0.06	–0.24**	0.05
BMI	–0.28***	0.08	–0.25**	0.06	–0.18*	0.03	–0.20**	0.04	–0.15*	0.02	–0.21**	0.04	–0.25**	0.06	–0.23**	0.05
BMI^2^	–0.28***	0.08	–0.26**	0.06	–0.18*	0.03	–0.20**	0.04	–0.15*	0.02	–0.22**	0.04	–0.25**	0.06	–0.23**	0.05
CRP	–0.28***	0.08	–0.25**	0.06	–0.17*	0.03	–0.19*	0.04	–0.15*	0.02	–0.21**	0.04	–0.26**	0.06	–0.23**	0.05
Cigarettes	–0.28***	0.07	–0.25**	0.06	–0.16*	0.02	–0.20*	0.04	–0.14	0.02	–0.20**	0.04	–0.25**	0.06	–0.23**	0.05
CES-D	–0.26***	0.07	–0.24**	0.05	–0.16*	0.03	–0.19*	0.03	–0.14	0.02	–0.20**	0.04	–0.25**	0.06	–0.22**	0.05
WMLV	–0.24***	0.05	–0.21**	0.04	–0.15*	0.02	–0.16*	0.02	–0.10	0.01	–0.16**	0.03	–0.23**	0.05	–0.21**	0.04


*SES = socioeconomic status; BMI = body mass index; CRP = C-reactive protein; CES-D = Center for Epidemiologic Studies-Depression scale; WMLV = white matter lesion volume; R = right; L = left. Demonstration of the main effects of SES with regional fractional anisotropy in the additional models that include a sensitivity variable. ^∗^*p* < 0.05, ^∗∗^*p* < 0.01, ^∗∗∗^*p* < 0.001. Data reflect standardized regression coefficients (β) and semipartial correlations squared (*sr^*2*^*). ^*a*^Base model includes SES, race, age, and sex.*

There were significant associations between whole-brain white matter lesion volume and FA in the bilateral frontal, parietal, and occipital lobes (all *p*’s < 0.05), but not the temporal lobes (*p*’s > 0.05). Adjustment for whole-brain white matter lesion volume attenuated the significant relation between SES and the R parietal lobe FA (attenuation to *p* = 0.169; Table 4). Adding whole-brain white matter lesion volume also rendered main effects of sex with the R frontal and bilateral temporal lobes significant (all *p*’s < 0.05), such that men had lower FA in these regions than women.

### Exploratory Analyses

There were no significant interactions between age, race, and the various independent SES indicators (i.e., poverty status, continuous education, and dichotomous education) on any of the cortical white matter FA regions (all *p*’s > 0.05). As displayed in Table 5, models with poverty status as the SES indicator yielded significant main effects for poverty status in the R frontal (β = -0.20, *p* = 0.006), L frontal (β = -0.21, *p* = 0.004), L parietal (β = -0.15, *p* = 0.042), R occipital (β = -0.16, *p* = 0.031), and L occipital (β = -0.17, *p* = 0.026) lobes, where individuals living in poverty had lower FA in these regions. A main effect for poverty status on the R parietal lobe was trending at β = -0.14, *p* = 0.052. In these models, age was related to all regions (β’s ranged from -0.21 to -0.28, all *p*’s < 0.01) except the R and L temporal lobes (*p*’s > 0.05), wherein older age was related to lower FA. A main effect for sex was found for the R parietal lobe, wherein men had lower FA in this region, β = -0.15, *p* = 0.031. There were no significant main effects for race (all *p*’s > 0.05). Models with dichotomous education as the SES indicator yielded a significant main effect for dichotomous education in the R occipital lobe (β = -0.17, *p* = 0.018). Significant main effects for age were found for nearly all regions (all *p*’s < 0.05), such that older age was associated with lower FA, with the exception of the R temporal lobe (β = -0.07, *p* = 0.319). No main effects for sex or race were identified in these models. Models with continuous education as the SES indicator resulted in no significant main effects for continuous education, race, or sex. With the exception of the R temporal lobe (β = -0.06, *p* = 0.418), significant main effects for age were found for all regions (all *p*’s < 0.05), such that older age was related with lower FA.

**Table 5:** SES indicators and regional fractional anisotropy: final models.

	SES Interaction Term (All *N*s = 192)					
Variable	Continuous Education		Dichotomous Education		Poverty Status	
	β	*sr^2^*	β	*sr^2^*	β	*sr^2^*
**R Frontal**						
Age	–0.27***	0.069	–0.27***	0.070	–0.28***	0.076
SES	0.12	0.014	–0.13	0.015	–0.20**	0.037
Race	–0.12	0.014	–0.13	0.017	–0.09	0.007
Sex	–0.10	0.009	–0.09	0.009	–0.11	0.013
**L Frontal**						
Age	–0.25**	0.060	–0.26**	0.062	–0.27***	0.070
SES	0.09	0.008	–0.11	0.012	–0.21**	0.045
Race	–0.11	0.011	–0.12	0.014	–0.07	0.005
Sex	–0.04	0.002	–0.04	0.002	–0.06	0.004
**R Temporal**						
Age	–0.06	0.003	–0.07	0.005	–0.08	0.006
SES	0.01	0.000	–0.09	0.008	–0.12	0.014
Race	–0.03	0.001	–0.05	0.002	–0.01	0.000
Sex	–0.11	0.011	–0.12	0.013	–0.13	0.015
**L Temporal**						
Age	–0.15*	0.021	–0.16*	0.023	–0.16	0.023
SES	0.09	0.007	–0.12	0.013	–0.12	0.012
Race	–0.02	0.000	–0.03	0.001	0.01	0.000
Sex	–0.11	0.012	–0.11	0.012	–0.12	0.013
**R Parietal**						
Age	–0.23**	0.051	–0.23**	0.051	–0.25**	0.059
SES	0.05	0.002	–0.03	0.001	–0.14^∧^	0.018
Race	0.02	0.001	0.02	0.000	0.05	0.002
Sex	–0.14	0.018	–0.13	0.018	–0.15*	0.023
**L Parietal**						
Age	–0.25**	0.058	–0.25**	0.058	–0.26***	0.064
SES	0.09	0.007	–0.08	0.006	–0.15*	0.021
Race	–0.05	0.003	–0.06	0.003	–0.03	0.009
Sex	–0.07	0.005	–0.07	0.004	–0.08	0.006
**R Occipital**						
Age	–0.25**	0.058	–0.26***	0.063	–0.26***	0.062
SES	0.10	0.010	–0.17*	0.028	–0.16*	0.023
Race	–0.07	0.005	–0.09	0.007	–0.04	0.002
Sex	–0.06	0.003	–0.06	0.003	–0.07	0.004
**L Occipital**						
Age	–0.20**	0.039	–0.21**	0.041	–0.21**	0.043
SES	0.10	0.009	–0.12	0.014	–0.17*	0.025
Race	–0.07	0.005	–0.08	0.007	–0.04	0.002
Sex	–0.10	0.009	–0.10	0.009	–0.11	0.012


SES = socioeconomic status; R = right; L = left. Final models reflect the models following the backward elimination procedure. ^∧^*p* = 0.05, *p < 0.05, **p < 0.01, ***p < 0.001. Data reflect standardized regression coefficients (β) and semipartial correlations squared (*sr^*2*^*).

There were no significant interactions between age, race, and the composite SES indicator on any of the trace variables. As displayed in Table 6, SES was related to trace in the R frontal, L frontal, R temporal, L temporal, and L occipital lobes, wherein individuals with low SES had higher trace values. There were significant main effects for age and sex on all regions (all *p*’s < 0.05) such that older individuals and men were more likely to have higher trace values. Relative to African Americans, Whites had higher trace values in the R and L parietal lobes.

**Table 6:** Sociodemographic variables and regional trace: final models.

	Outcome (Trace; *N* = 192)															
	R Frontal		L Frontal		R Temporal		L Temporal		R Parietal		L Parietal		R Occipital		L Occipital	
Predictor	β	*sr^2^*	β	*sr^2^*	β	*sr^2^*	β	*sr^2^*	β	*sr^2^*	β	*sr^2^*	β	*sr^2^*	β	*sr^2^*
Age	0.52***	0.252	0.53***	0.263	0.38***	0.132	0.34***	0.106	0.45***	0.187	0.46***	0.200	0.41***	0.155	0.41***	0.154
SES	0.20**	0.036	0.16*	0.023	0.19**	0.032	0.16*	0.022	0.07	0.005	0.11	0.011	0.12	0.014	0.20**	0.035
Race	–0.02	0.000	–0.05	0.003	–0.17*	0.027	–0.10	0.009	–0.20**	0.038	–0.15*	0.020	–0.10	0.009	0.07	0.004
Sex	0.26***	0.064	0.24***	0.057	0.17**	0.029	0.23**	0.050	0.32***	0.096	0.30***	0.086	0.22**	0.045	0.22**	0.045


SES = socioeconomic status; R = right; L = left. Final models reflect the models following the backward elimination procedure. *p = 0.05, **p < 0.01, ***p < 0.001. Data reflect standardized regression coefficients (β) and semipartial correlations squared (*sr^*2*^*).

## Discussion

To our knowledge, this is the first study to examine independent and interactive relations between SES, race, and age with white matter integrity in primary cortical regions. Our findings demonstrate sociodemographic disparities in the brain’s white matter microstructure, which may have implications for cognitive, functional, and neurological disease-related outcomes. Although no significant interactions were observed, our results suggest poorer diffuse white matter integrity, on average, for individuals of lower SES. There were no differences in white matter integrity between racial groups and, as expected, poorer white matter integrity was associated with older age. When examining additional SES indicators, poverty status revealed as most prominently related to SES. Further exploratory analyses showed greater diffusion (captured via trace) in individuals with lower SES in the R and L frontal, R and L temporal, and L occipital lobes. Greater diffusion throughout the brain was also associated with older age and male sex, as well as with being White for the parietal lobes.

These results add to the limited literature demonstrating an SES-white matter integrity association in community-dwelling adults. As far as we are aware, this is the first study to demonstrate that the SES-white matter integrity link appears to be uniform across the brain’s primary cortical regions, as opposed to differentially across particular lobes or hemispheres. The relation between SES and trace, which can be used to measure alterations in brain tissue (Bosch et al., 2012), was also fairly widespread, with the temporal lobes and R occipital lobe spared. These findings add to studies demonstrating an anterior-posterior diffusion gradient (Sullivan and Pfefferbaum, 2006; Bosch et al., 2012), as well as increased diffusivity in occipital regions in vulnerable clinical populations (Friese et al., 2010). These findings are consistent with prior literature finding a link between lower SES and compromised white matter integrity in children (Ursache and Noble, 2016; Dufford and Kim, 2017) and adults (Teipel et al., 2009; Piras et al., 2011; Gianaros et al., 2013; Takeuchi et al., 2018). These studies, however, focused on individual tracts, whereas this study examined these associations from a regional lobar perspective. Both approaches are vital for better understanding SES-white matter relations.

The association of lower SES with poorer diffuse white matter integrity is important given the adverse cognitive outcomes associated with reduced white matter integrity. There is ample evidence from clinical and normal aging samples demonstrating “disconnection syndromes,” wherein compromised microstructure of the white matter tracts is thought to lead to poorer communication between brain regions, ultimately resulting in poorer cognitive function and more profound cognitive decline (O’Sullivan et al., 2001; Chanraud et al., 2010). This is relevant given the well-established literature demonstrating poorer performance, on average, on tests of cognitive function across a range of domains in individuals from lower SES homes (e.g., Singh-Manoux et al., 2005; Noble et al., 2007; Hackman and Farah, 2009; Shaked et al., 2018). While not examined directly here, perhaps SES-related differences on cognitive tests are, at least in part, explained by disparities in diffuse white matter integrity. Future studies should examine the potential mediating role of white matter integrity in the relation between SES and cognitive outcomes. Moreover, white matter microstructural properties (e.g., white matter integrity) are considered a proxy for brain reserve (Stern et al., 2018), which is defined as “neurobiological capital…that allows some people to better cope with brain aging and pathology than others (p. 3, Stern et al., 2018).” Within this context our findings of altered white matter integrity suggest that individuals from lower SES homes have lower levels of such reserve. Future studies should assess if less of this form of reserve puts low SES individuals at greater risk for more profound age- and disease-related changes, such as steeper cognitive decline in the face of Alzheimer’s disease pathology.

Exploratory analyses showed that poverty status and education were differentially related to white matter integrity across the examined brain regions. Prior literature has noted that different socioeconomic influences play varying roles in brain plasticity (Farah, 2017). Poverty status seems to represent widespread correlates of economic status, such as material resources and financial hardships, as well as nutrition and toxin exposure. While also a proxy for resources and opportunity, educational attainment may better capture school-related factors like language stimulation and literacy (although quantity of formal education is not equivalent to education quality). The lack of findings with education alone is notable given the literature demonstrating relations between educational attainment and white matter integrity (Teipel et al., 2009; Chiang et al., 2011; Gianaros et al., 2013; Johnson et al., 2013; Noble et al., 2013). One potential reason for these discrepant findings is that some studies (Chiang et al., 2011; Johnson et al., 2013) used composite indices, and it is therefore unknown if education was the primary driver in those studies’ findings. Another possible explanation is the nature of the study samples. Our sample was comprised of a sociodemographically diverse group of urban-dwelling adults, while one study was conducted in children (Noble et al., 2013), and others had no (Teipel et al., 2009) or a smaller proportion of (Gianaros et al., 2013) African Americans in their sample. Given that poverty status captured more of the findings than education alone in our study, perhaps relative to educational attainment, poverty status is a greater influencer of disparities in white matter integrity in this socioeconomically and racially diverse sample of adults. That said, given that the SES composite revealed more significant findings and larger effect sizes than findings with poverty status alone (see Tables 3, 5), perhaps for adults, the cumulative nature of education and poverty status is a relatively stronger determinant of disparities in white matter integrity.

While it is evident that there are individual differences in white matter health across SES groups, the biopsychosocial factors that are most important for this variability are not well established. It is known, for instance, that cardiovascular risk factors like hypertension and cigarette smoking adversely impact white matter integrity (e.g., Gianaros et al., 2013; Wang et al., 2015), but other factors like depression and stress have equivocal results (Gianaros et al., 2013; Choi et al., 2014; Hermens et al., 2018). These inconsistent findings are perhaps surprising given the well-established relations of stress and depression on inflammation, which is closely related to white matter integrity (Wersching et al., 2010; Gianaros et al., 2013; Walker et al., 2017).

The sensitivity analyses found that adjustment of several common cardiovascular and inflammatory risk factors did not attenuate significant effects of SES on FA of the primary cortical regions, nor were they significantly related to FA in these regions. The lack of attenuation could be due to the absence of relations between SES and most of the health variables (Table 1). These findings are perhaps counter-intuitive given the literature demonstrating disparities in cardiovascular and inflammatory risk factors across socioeconomic groups (Adler et al., 1994; Pollitt et al., 2005). However, they may also reflect prior literature demonstrating complex interactive relations of SES, race, and/or gender with respect to cardiovascular risk factors (Williams et al., 2010; Waldstein et al., 2016). In that regard, it has been noted across multiple investigations that higher SES African Americans often have worse cardiovascular risk profiles than lower SES African Americans, perhaps reflecting the “diminishing returns” hypothesis (Farmer and Ferraro, 2005), which posits that African Americans may not benefit as much as Whites from higher levels of SES.

Although it is possible that other vascular and biomedical risk factors are implicated in the observed SES-white matter associations, it is also possible that adjustment for individual risk factors does not fully capture the cumulative burden of vulnerability associated with lower SES across the lifespan. Research has shown an association between early life adversity and lower adult SES (Metzler et al., 2017), and that children of lower SES, on average, have poorer white matter integrity than children of higher SES (Ursache and Noble, 2016; Dufford and Kim, 2017), although results have been inconsistent across studies (Chiang et al., 2011; Jednorog et al., 2012). This suggests that SES-related differences in FA may begin in childhood, and perhaps, continue to widen due to lifelong exposure to various biopsychosocial risk factors, including inadequate nutrition (Hartline-Grafton and Dean, 2017), lesser access to health care (Lazar and Davenport, 2018), chronic stress (Baum et al., 1999), environmental toxins (Evans and Kantrowitz, 2002), and greater overall burden of disease (Pathirana and Jackson, 2018).

Adjustment for depressive symptoms, cigarette smoking, and whole-brain white matter lesion volume negated associations between SES and FA in the R parietal lobe. The size of the SES effect however was largely unchanged following adjustment for depressive symptoms and cigarette smoking (*sr^2^* = 0.02, with and without adjustment for these variables; see Table 4). Previous DTI research has demonstrated that major depressive disorder is significantly associated with decreased FA in left hemisphere regions (Zou et al., 2008), consistent with other neuroimaging research suggesting a left-hemisphere dominance for symptoms of depression (Morris et al., 1996). The lack of relation between depressive symptoms and left hemisphere regions may be due to not assessing for a validated diagnosis of major depressive disorder, but rather depressive symptom severity. More research is needed to identify psychosocial and behavioral risk factors that are associated with reduced FA, and how they operate within the mechanistic pathways by which lower SES adversely relates to white matter structure.

Following adjustment for whole-brain white matter lesion volume, the size of the SES effect on the R parietal lobe reduced from *sr^2^* = 0.02 to *sr^2^* = 0.01 (Table 4). Although small, this reduction may suggest that overall maturation and/or white matter vascular burden is implicated in SES-related FA differences in this region. That said, significant associations between SES and FA in other cortical white matter regions remained significant, indicating that SES-related difference in white matter integrity throughout the brain exist above and beyond overall white matter maturation. It is worth noting that out of all the cortical regions examined, the R parietal lobe, while significant, had the weakest relation with SES in the base models. The relation between SES and the R parietal lobe may therefore not be strong enough to maintain significance following the loss of power.

Consistent with past research (Salat et al., 2005), the present study found that older age was associated with significantly lower FA and higher trace throughout the brain, with the exception of the R temporal lobe for FA. It is possible that in an older sample, greater age would also be associated with lower R temporal lobe FA, or that further region-specific differences would be observed. A previous study reported that higher SES (versus lower SES) was associated with greater white matter integrity in three frontal tracts in older (i.e., 66 years), but not younger (i.e., 33 years) participants (Johnson et al., 2013). The present study’s finding that age did not moderate associations between SES and white matter integrity may therefore reflect our relatively younger sample. It is also possible that differences in SES measurement accounted for these differences. The previous study used a composite of educational attainment and occupation, whereas our study used a composite of educational attainment and poverty status. Indeed, SES is a multidimensional construct (Braveman et al., 2005), and different socioeconomic indicators may be differentially associated with brain outcomes. Interestingly, widespread associations were also found between sex and trace, where relative to women, men had greater levels of diffusion throughout the entire brain. This is consistent with a recent study of young adults (*N* = 1,216) finding a significant sex by SES interaction, where among those with higher levels of family income and level of education, men had higher mean diffusivity (an average of trace) relative to women (Takeuchi et al., 2018). Perhaps with a larger sample size and greater statistical power, our study would have produced similar results. Future studies should seek to determine mediators and additional moderators of the trace-sex relation, as to better understand why men are vulnerable to greater diffusivity, but not poorer white matter integrity.

Given that the literature on racial differences in white matter microstructure is limited, our study, which found non-significant independent and moderating effects of self-identified race with FA in a community-dwelling sample, represents a novel contribution to the literature. The lack of significant interactive relations between race and SES is surprising, given previous findings in the HANDLS SCAN sample demonstrating such an interaction with white matter lesion volume (Waldstein et al., 2017) and volumes of stress-related brain regions (Shaked et al., 2019). Further, irrespective of SES, African Americans experience greater burden of stroke (Morgenstern et al., 1997), white matter lesions (Liao et al., 1997), and dementia than their White counterparts (Mayeda et al., 2016), as well as exposure to biomedical (e.g., hypertension, diabetes mellitus), psychosocial (e.g., social discrimination, chronic stress), and environmental (e.g., geographic segregation, toxin exposure) risk factors that influence brain and cognitive health across the lifespan (Glymour and Manly, 2008; Williams et al., 2010). One possibility for the null race-related findings is that social risk factors specifically linked to self-identified race, such as racial discrimination, are unrelated to white matter integrity, but exert influence on global and regional brain and white matter lesion volumes. This possibility, however, is highly speculative and needs to be examined directly in future studies. Unexpectedly, relative to African Americans, White individuals had greater diffusion in the parietal lobes. One explanation for these findings is that Whites are significantly older than the African Americans in our sample (*p* = 0.03, Whites = 53 years, African Americans = 50 years). As noted, age was strongly related to greater diffusion (Table 6; βs ranged from 0.34 to 0.53), and the race effects may be due to residual confounding of age. Further research on biopsychosocial factors that are uniquely associated with white matter integrity and diffusivity could help clarify potential race differences in DTI indices.

This study had notable strengths. The HANDLS investigation was explicitly designed to disentangle SES- and race-related health disparities, and therefore our present study contained a wide range of SES among African American and White community-dwelling adults. Our study used a SES composite comprised of two key indicators, educational attainment and poverty status, which are implicated in brain health and aging. This was the first study to examine potential moderating effects of race and age on the association between SES and FA in key cortical regions and expanded on previous research by examining how further adjustment of relevant biopsychosocial risk factors changed associations of SES and FA.

This study also had several limitations. The results may be specific to adults living within the urban environment of Baltimore City. Future studies should examine associations of sociodemographic factors with FA in other racial/ethnic minorities and participants living in non-urban environments. Moreover, while our imaging subsample was representative of the larger HANDLS sample with regards to poverty status, years of education, and sex, the imaging subsample was significantly more likely to include younger and White participants. Conclusions regarding race and age effects should therefore be generalized with caution to the overall HANDLS sample. Also, while we examined the influence of several cardiovascular risk factors, we did not account for the duration of illnesses, influences of different classes of medication, and medication adherence, as HANDLS did not collect this information. Our composite measure of SES did not assess other socioeconomic indicators, such as occupational status, wealth, or income. Future studies should evaluate how additional SES indicators influence white matter microstructure in a socioeconomically diverse sample of adults. Also, we did not directly examine outcome variables such as cognitive ability and decline, which are important when considering the functional implications of our findings. Future studies should examine the potential mediating role of DTI outcomes in SES disparities of cognition and other functional outcomes related to white matter integrity. Finally, our study was cross-sectional and therefore did not examine within-subject age-related changes in white matter integrity. Longitudinal studies should examine how SES and self-identified race predict age-related degradation in FA.

In sum, lower SES was associated with poorer white matter integrity uniformly across the brain’s primary cortical regions, and with greater diffusion in the R and L frontal, R and L temporal, and L occipital lobes. These findings may reflect, at least in part, disproportionate exposure to biopsychosocial risk factors among those of lower SES, and may translate into more pronounced risk for age- and/or disease-related cognitive decline. Subsequent adjustment of vascular and inflammatory risk factors did not result in attenuation of SES effects anywhere in the brain, although depressive symptoms, smoking status, and white matter lesion volume negated the relation between SES and the R parietal lobe. Consistent with previous research, older age was also associated with poorer white matter integrity and greater diffusivity throughout the primary cortical regions. No differences in white matter integrity were found between self-identified African Americans and Whites, nor did our findings reveal significant interactions among SES, race, and age with white matter integrity in any region. The profound differences in white matter microstructure across SES groups is very relevant given the adverse cognitive, functional, and neurological-disease outcomes associated with poorer white matter integrity. Ideally, this research will encourage researchers and society at large to promote brain health across the socioeconomic spectrum. Future research is needed to identify mechanistic determinants of the SES-white matter relation as to promote targeted interventions and prevention efforts.

## Data Availability

Data are available upon request to researchers with valid proposals who agree to the confidentiality agreement as required by our Institutional Review Boards. We publicize our policies on our website^2^. Requests for data access may be sent to AZ (co-author) or the study manager, Jennifer Norbeck at norbeckje@mail.nih.gov.

## Ethics Statement

This study was carried out in accordance with the recommendations of the University of Maryland, Baltimore and the University of Maryland, Baltimore County Institutional Review Boards with written informed consent from all subjects. All subjects gave written informed consent in accordance with the Declaration of Helsinki.

## Author Contributions

DS, DL, and SW: general conception. ME, AZ, and SW: parent study design. DS and DL: data analysis and drafting the manuscript. DS, DL, LK, CD, RG, SS, GE, ME, AZ, and SW: final preparation of the article, data collection, preparation, and interpretation.

## Conflict of Interest Statement

The authors declare that the research was conducted in the absence of any commercial or financial relationships that could be construed as a potential conflict of interest.

## Footnotes

1 http://www.questdiagnostics.com

2 https://handls.nih.gov/
